# Efficacy of olmesartan amlodipine in Colombian hypertensive patients (soat study)

**DOI:** 10.1186/s13104-017-2486-z

**Published:** 2017-04-26

**Authors:** Richard Buendia, Monica Zambrano

**Affiliations:** 1 Colsubsidio Centro de Especialistas, Pontificia Universidad Javeriana, Hospital de la Policía, Bogotá, D.C. Colombia; 2 Colsubsidio Centro de Especialistas, Pontificia Universidad Javeriana, Hospital de la Samaritana, Bogotá, D.C. Colombia

**Keywords:** Olmesartan, Amlodipine, Practice patterns

## Abstract

**Background:**

Emerging evidence has shown a significant deficit in the control of hypertension (blood pressure <140/90 mmHg) among Hispanics or Latinos in about 65%. This study aims to determine the efficacy of the combination in fixed doses of olmesartan and amlodipine (20/5, 40/5, and 40/10 mg) in hypertensive patients treated in daily clinical practice by Colombian doctors.

**Methods:**

This was an observational, retrospective, open-label, multi-center, non-comparative study. The primary outcome was a change in systolic and diastolic blood pressure from the baseline to week 12; the secondary outcome was the proportion of patients achieving a target blood pressure of <140/90 mmHg. Safety and tolerability were also evaluated. For analysis, a student *t* test was used for paired data, McNemar test, and ANCOVA.

**Results:**

A total of 428 patients were enrolled from 16 centers in Colombia. At 12 weeks, patients’ systolic blood pressure decreased in response to all three doses: by 27.75 ± 20.73 mmHg in 20/5 mg, 31.13 ± 22.23 mmHg in 40/5 mg, and 46.96 ± 20.15 mmHg in 40/10 mg (all p < 0.001). Furthermore, the diastolic blood pressure decreased by 14.19 ± 12.89 mmHg in 20/5 mg, 16.25 ± 10.87 mmHg in 40/5 mg, and 24.83 ± 10.41 mmHg in 40/10 mg (all p < 0.001). The percentage of patients achieving target blood pressure was 71.31% in 20/5 mg, 70.16% in 40/5 mg, and 63.33% in 40/10 mg.

**Conclusions:**

This study demonstrates the efficacy of the combination in fixed doses of olmesartan and amlodipine in the treatment of Colombian hypertensive patients.

## Background

Hypertension is a highly prevalent disease that ranges from 30 to 44% and remains under diagnosed and undertreated [1–3]. Highly effective therapies are therefore required to avoid complications in target organs such as the retina, kidney, brain, and heart. Emerging data seem to suggest that Hispanics might have rates of uncontrolled hypertension that significantly exceed the rates observed in non-Hispanic people [4]. The Hispanic Community Health Study/Study of Latinos is a longitudinal cohort study of 16,415 Hispanics/Latinos. This study shows a significant deficit in the control of hypertension (blood pressure <140/90 mmHg) among Hispanics/Latinos in about 65% [5]. The combination of olmesartan and amlodipine in fixed doses (FDC) could be an effective choice for the management of hypertension in Hispanic patients. This study aims to determine the efficacy of the combination of olmesartan and amlodipine FDC in Colombian hypertensive patients for the reduction of systolic and diastolic blood pressure values; it also aims to determine the adverse effects associated with such treatment.

## Methods

This study was an observational, open-label, retrospective, multi-center, non-comparative clinical study with a total treatment period of 12 weeks, conducted at 16 clinical sites in Colombia. The cities included in the study were Barranquilla, Santa Marta, Cartagena, Monteria, Valledupar, Medellín, Bucaramanga, Cúcuta, Bogotá, Villavicencio, Ibagué, Neiva, Cali, Pereira, Armenia, and Ipiales.

The sample size was calculated seeking a reduction of 20 mmHg of systolic blood pressure (SBP) and 10 mmHg of diastolic blood pressure (DBP) with the use of olmesartan and amlodipine, with a p value <0.05 and a power of 80%, resulting in at least 246 patients.

The inclusion criteria encompassed patients over 18 years old, untreated patients with uncontrolled hypertension (≥140/90 mmHg), creatinine clearance >60 ml/min or creatinine <1.3 mg/dl, with or without other cardiovascular risk factors, who signed the informed consent and had no contraindication for the use of olmesartan and/or calcium antagonists.

The patients with any of these conditions such as: secondary hypertension, hypertensive emergency, or target organ damage such as heart failure, unstable angina, hypertensive encephalopathy, myocardial infarction, stroke, history of coronary revascularization, valvular heart disease, and serious arrhythmia. Patients with severe organ dysfunction, as assessed by laboratory abnormality, and pregnancy were excluded.

The blood pressure data was collected from the medical records of clinical sites and included baseline, 6, and 12 weeks, according to the combination of olmesartan and amlodipine fixed doses of 20/5, 40/5, and 40/10 mg.

The data was processed by the analysis of paired observations, paired student t-test, and the McNemar test for categorical data analysis. The SBP and DBP at weeks 0, 6, and 12 were analyzed using the repeated-measures analysis of covariance (ANCOVA) with covariates of baseline SBP and DBP. Two-tailed p values of less than 0.05 were considered to be statistically significant. Statistical analyses were conducted by using STATA 12 (StataCorp).

The primary outcome was a change in SBP and DBP from the baseline to week 12. The secondary outcome was the proportion of patients achieving a target blood pressure of <140/90 mmHg. The reduction of pulse pressure (PP) was defined as the difference between systolic and diastolic pressure for each dose of olmesartan and amlodipine FDC.

The blood pressure (BP) was measured with mercury sphygmomanometers that were appropriate for each patient’s anthropometric parameters, and both sides of the arms were measured. The BP used in the study was the mean of three measurements, which were performed after 10 min of rest in the seated position. The BP was read in 2 mmHg intervals to prevent the risk of bias.

The study was conducted according to the Good Clinical Practice guidelines and the Declaration of Helsinki.

## Results

A total of 428 patients were enrolled from 16 centers in Colombia. Of these patients, 46.96% were male, 3.27% were diabetic, 42.7% received olmesartan and amlodipine FDC 20/5 mg, 46.2% received a dose of 40/5 mg, and 10.9% received a dose of 40/10 mg. The mean age was 61.35 ± 12.72 years; the SBP mean was 162.04 ± 18.88 mmHg, and the DBP mean was 96.36 ± 10.53 mmHg.

**Fig. 1:**
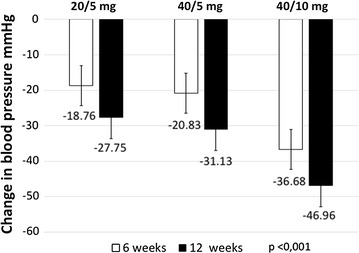
Reduction of mean systolic blood pressure values at 6 and 12 weeks for each dose of olmesartan and amlodipine FDC

Patients with hypertension stage 1 accounted for 30.84% of the sample, while patients with hypertension stage 2 comprised 69.16%, according to criteria of the Joint National Committee (JNC 8) [6] (see Table 1). The SBP at 6 weeks showed a statistically significant reduction from baseline values in all three groups of olmesartan and amlodipine FDC. With a dose of 20/5 mg, the SBP was 18.76 ± 21.72 mmHg; with a 40/5 mg dose, SBP was 20.83 ± 22.64 mmHg; and with a 40/10 mg dose, SBP was 36.68 ± 21.87 mmHg (all p < 0.001). At 12 weeks, the SBP decreased in all three doses: by 27.75 ± 20.73 mmHg in 20/5 mg, 31.13 ± 22.23 mmHg in 40/5 mg, and 46.96 ± 20.15 mmHg in 40/10 mg (all p < 0.001). There were statistically significant differences between dose groups 20/5 mg and 40/5 mg, p < 0.001; 20/5 mg and 40/10 mg, p = 0.036; however, there were no differences between the doses 40/5 mg and 40/10 mg, p = 1 (see Table 2; Fig. 1).

**Table 1:** Baseline characteristics

Category	N	%
Sex		
Male	201	46.96
Female	227	53.04
Diabetes mellitus	14	3.27
Hyperlipidemia	38	8.88
Olmesartan and amlodipine doses		
20/5 mg	183	42.76
40/5 mg	198	46.26
40/10 mg	47	10.98
Hypertension stage		
Hypertension stage 1	132	30.84
Hypertension stage 2	296	69.16

Numeric variables	Mean	SD
Systolic blood pressure	162.04	18.88
Diastolic blood pressure	96.36	10.53
Pulse pressure	65.67	16.81
Age	61.35	12.72
Total Cholesterol mg/dl	210.46	39.47
LDL Cholesterol mg/dl	115.39	41.06
HDL Cholesterol mg/dl	47.04	14.21
Triglycerides mg/dl	181.86	93.25
Hemoglobin A1c %	6.36	0.84
Creatinine	0.9	0.19

N = 428 patients

**Table 2:** Reduction of mean systolic blood pressure values at 6 and 12 weeks for each dose of olmesartan and amlodipine FDC

Olmesartan and amlodipine doses	Baseline (mean ± SD)	6 weeks (mean ± SD)	Difference initial/6 weeks (mean ± SD)	p	12 weeks (mean ± SD)	Difference initial/12 weeks (mean ± SD)	p
20/5 mg (N = 183 patients)	157 ± 16.28	138.45 ± 14.10	18.76 ± 21.72	<0.001	129.24 ± 12.92	27.75 ± 20.73	<0.001
40/5 mg (N = 198 patients)	161.93 ± 17.92	140.85 ± 14.04	20.83 ± 22.64	<0.001	130.79 ± 10.77	31.13 ± 22.23	<0.001
40/10 mg (N = 47 patients)	176.46 ± 18.68	140 ± 12.88	36.68 ± 21.87	<0.001	129.5 ± 12.40	46.96 ± 20.15	<0.001

N = 428 patients

**Fig. 2:**
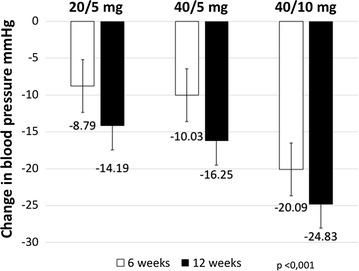
Reduction of mean diastolic blood pressure values at 6 and 12 weeks for each dose of olmesartan and amlodipine FDC

The DBP values at 6 weeks also showed a statistically significant decrease from the baseline in all three groups of olmesartan and amdolipine FDC: with a 20/5 mg dose, DBP was 8.79 ± 12.85 mmHg; with a 40/5 mg, DBP was 10.03 ± 10.67 mmHg; and with a 40/10 mg dose, DBP was 20.09 ± 12.44 mmHg (all p < 0.001). At 12 weeks, the reduction with a 20/5 mg dose was 14.19 ± 12.89 mmHg; with a 40/5 mg dose, the reduction was 16.25 ± 10.87 mmHg, and with a 40/10 mg, the reduction was 24.83 ± 10.41 mmHg (all p < 0.001). There was no difference between dose groups of 20/5 and 40/5 mg (p = 0.95), 20/5 mg and 40/10 mg (p = 0.108), and 40/5 mg and 40/10 mg (p = 0.33) (see Table 3; Fig 2).

**Table 3:** Reduction of mean diastolic blood pressure values at 6 and 12 weeks for each dose of olmesartan and amlodipine FDC

Olmesartan and amlodipine doses	Baseline (mean ± SD)	6 weeks (mean ± SD)	Difference initial/6 weeks (mean ± SD)	p	12 weeks (mean ± SD)	Difference initial/12 weeks (mean ± SD)	p
20/5 mg (N = 183 patients)	93.44 ± 10.20	84.76 ± 7.79	8.79 ± 12.85	<0.001	79.24 ± 7.89	14.19 ± 12.89	<0.001
40/5 mg (N = 198 patients)	96.91 ± 7.98	86.68 ± 8.72	10.03 ± 10.67	<0.001	80.66 ± 6.67	16.25 ± 10.87	<0.001
40/10 mg (N = 47 patients)	103.26 ± 7.77	83.59 ± 8.22	20.09 ± 12.44	<0.001	78.43 ± 6.07	24.83 ± 10.41	<0.001

N = 428 patients

The SBP mean of the diabetic patients was 153.36 ± 32.24 mmHg (range: 120-230) and in patients with dyslipidemia the SBP mean was 154.29 ± 25.23 mmHg (range: 70–230). The DBP mean in diabetic patients was 83.84 ± 15.56 mmHg (range: 60–120) and patients with dyslipidemia was 94.82 ± 11.67 mmHg (range 65–120).

The reduction in SBP in diabetic patients was 37.12 mmHg (p = 0.072) at 6 weeks and 39.12 mmHg (p = 0.032) at 12 weeks. In hyperlipidemic patients SBP was reduced by 26.64 mmHg (p = 0.45) at 6 weeks and 26.64 mmHg (p = 0.89) at 12 weeks.

The reduction in DBP in diabetic patients was 16.43 mmHg (p = 0.064) at 6 weeks and 24.83 mmHg (p = 0.001) at 12 weeks, in patients with hyperlipidemia DBP was reduced by 27.38 mmHg (p < 0.001) at 6 weeks and 32.38 mmHg (p = 0.002) at 12 weeks.

**Fig. 3:**
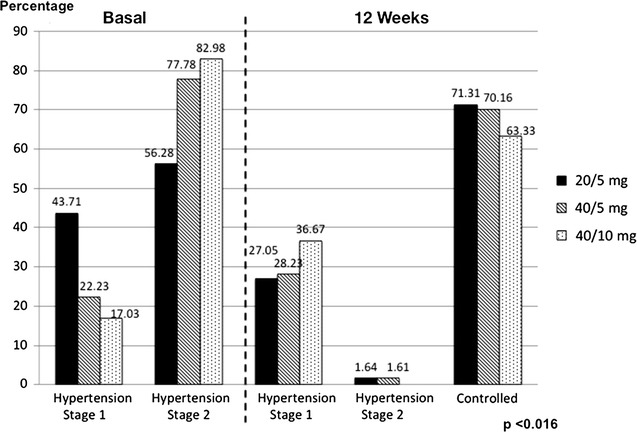
Dosage prescribed of olmesartan and amlodipine FDC according to stage of hypertension, baseline and 12 weeks

The dosage of olmesartan and amlodipine FDC prescribed in hypertension stage 1 was 43.71% in 20/5 mg, 22.23% in 40/5 mg, and 17.03% in 40/10 mg. In stage 2, the dosage was 56.28% in 20/5 mg, 77.78% in 40/5 mg, and 82.98% in 40/10 mg, these proportion of hypertensive patients stage 2 decrease statistically significant to 1.64% with the dose of 20/5 mg, 1.61% in 40/5 mg, and 0% in 40/10 mg at 12 weeks of olmesartan and amlodipine FDC (p < 0.016) (Fig. 3).

The secondary outcome was the proportion of patients achieving a target BP of <140/90 mmHg (controlled). At 12 weeks, 71.31% of patients with a dose of 20/5 mg, 70.16% with a dose of 40/5 mg, and 63.33% with a dose of 40/10 mg had achieved the target (p < 0.016). There were no differences by sex (p = 0.19) or among diabetic (p = 0.625) or hyperlipidemic (p = 0.203) patients (Fig. 3).

The pulse pressure at 12 weeks demonstrated a statistically significant reduction with each dose of olmesartan and amlodipine FDC. The dose of 20/5 mg in 13.55 ± 10.17 mmHg, with 40/5 mg in 14.88 ± 21.29 mmHg, and with 40/10 mg in 22.13 ± 16.78 mmHg (all p < 0.001) (see Table 4).

**Table 4:** Reduction of mean pulse pressure values at 12 weeks for each dose of olmesartan and amlodipine FDC

Olmesartan and amlodipine doses	Baseline (mean ± SD)	12 weeks (mean ± SD)	Difference initial/12 weeks (mean ± SD)	p
20/5 mg (N = 183 patients)	63.55 ± 15.57	50 ± 11.19	13.55 ± 18.84	<0.001
40/5 mg (N = 198 patients)	65.01 ± 16.92	50.12 ± 10.54	14.88 ± 21.29	<0.001
40/10 mg (N = 47 patients)	73.2 ± 17.09	51.06 ± 11.21	22.13 ± 16.78	<0.001

N = 428 patients

There was no report of discontinuation of treatment with the use of olmesartan and amlodipine FDC for 12 weeks. The proportions of adverse effects were as follows: headache, 4 patients (0.9%); dizziness, 5 patients (1.17%); and peripheral edema; 3 patients (0. 7%).

## Discussion

In this 12 week study, we found that the olmesartan and amlodipine FDC was effective in controlling systolic and diastolic BP in clinical practice for Colombian patients. There was a statistically significant reduction in systolic blood pressure values after 6 weeks of treatment, between 18 and 36 mmHg, depending on the dose received; the reduction after 12 weeks was between 26 and 47 mmHg. The diastolic blood pressure values were also reduced, between 8 and 20 mmHg at 6 weeks and between 14 and 24 mmHg at 12 weeks.

These results are higher than those found in pivotal studies such as the COACH study (The factorial Combination of olmesartan medoxomil and amlodipine besylate in Controlling High Blood Pressure)—a randomized, placebo-controlled study in which the efficiency of dual combination therapy with olmesartan and amlodipine was compared with its components in monotherapy. In patients with hypertension from mild to severe, reductions in SBP values were found between 23 and 30 mmHg; DBP values were reduced between 14 and 19 mmHg [7]. Volpe et al. [8] conducted a randomized, multi-center, single-arm study of 755 patients with hypertension in monotherapy with amlodipine 5 mg/day without response. They were then randomized to olmesartan and amlodipine FDC 20/5, 40/5, and 40/10 mg. The reduction in systolic and diastolic blood pressure values were found to be in the range of 16.8 mmHg to 9.6, respectively (p < 0.0001). In the study BP-CRUSH [9] open-label, multi-center and single-arm, with olmesartan and amlodipine plus hydrochlorothiazide if necessary to maintain a blood pressure of 120/70 mmHg—at 20 weeks, the study showed reductions in systolic blood pressure values between 14 and 20.3 mmHg and in diastolic values between 7.7 and 11.3 mmHg (p < 0.001).

In patients with diabetes and hyperlipidemia, there are interesting results in reducing blood pressure levels with the combination olmesartan and amlodipine FDC, although caution should be exercised in interpreting the results, because of the reduced proportion of patients with diabetes (3.27%) and hyperlipidemia (8.88%) in the study.

Interestingly, the dosage of olmesartan and amlodipine FDC prescribed was directly proportional to the stage of hypertension; for example, the dose of 40/10 mg was prescribed more in stage 2, while a 20/5 mg dose was prescribed in stage 1. The proportion of hypertensive patients in stage 2 at the baseline (between 56.28 and 82.98, depending on the dose of olmesartan and amlodipine FDC) decreased statistically significant between 0 and 1.64% at 12 weeks (p < 0.016), demonstrating a high antihypertensive efficacy of different doses of olmesartan and amlodipine FDC.

In the secondary outcome, this study showed a significant proportion of patients achieving their blood pressure goal (<140/90 mmHg) with olmesartan and amlodipine FDC. Between 63 and 71% showed trends similar to previous studies such as COACH (between 42.5 and 51%) [7] and BP-CRUSH (between 49 and 77%) [9].

Recently, it was found that Latin American patients with high pulse pressure are associated with 2.6 times more likely to have cardiovascular events [10], and in American patients pulse pressure has been associated with increased overall mortality causes and cardiovascular mortality, from 29 to 54% [11]. A meta-analysis of 14 studies with 510.546 patients found that an increase in brachial pulse pressure of 10 mmHg was associated with an increase of 13% in cardiovascular events and 9% in mortality [12].

This study found an important and significant reduction in pulse pressure of 13–22.13 mmHg. In the light of the evidence, this could reduce morbidity and mortality in our patients, but another type of study would be required to determine its effect.

Increased blood pressure is an important risk factor for stroke, heart disease, and kidney failure. Many clinical trials have shown that reducing the BP by a variety of strategies reduces the risk of stroke by about 35%, congestive heart failure by 42%, and coronary heart disease by 28% [13–15]. The European guidelines for hypertension recommend a target SBP and DBP of <140/90 mmHg in the general population [16, 17]. In these guidelines, blockers—angiotensin receptor and calcium channel blockers—are recommended as a first-line treatment, either as monotherapy or in combination.

The limitations of this study are mainly due to its retrospective, open-label design, short study duration, and the possible underreporting of adverse effects. The evidence has shown a range of adverse effects with olmesartan and amlodipine, such as edema (in 0.5%), headache, and dizziness (in 1.6% on average) [8].

In Colombia, the prevalence of hypertension is 23%, of which 59.8% are diagnosed, 36.3% are being treated and only 11.8% are correctly controlled [18], therefore in our country, hypertension is a public health problem.

The strength of this study is that it is a multi-center study with Colombian representative populations; it provides the first evidence of the effectiveness of olmesartan and amlodipine in daily clinical practice in our country.

## Conclusion

In this study of daily clinical practice in Colombian patients, the FDC of olmesartan/amlodipine provides excellent systolic and diastolic blood pressure control. More than 63% of patients achieved their blood pressure goal—reduced pulse pressure of 13 mmHg—and the medication combination is well tolerated.

## Abbreviations

SBP: systolic blood pressure
DBP: diastolic blood pressure
BP: blood pressure
FDC: combination in fixed doses
PP: pulse pressure
